# Development and Validation of HPLC Method for Determination of Crocetin, a constituent of Saffron, in Human Serum Samples

**Published:** 2013-01

**Authors:** Amir Hooshang Mohammadpour, Mohammad Ramezani, Nasim Tavakoli Anaraki, Bizhan Malaekeh-Nikouei, Sara Amel Farzad, Hossein Hosseinzadeh

**Affiliations:** 1 *Pharmaceutical Research Center, Department of Pharmacodynamics and Toxicology, School of Pharmacy, Mashhad University of Medical Sciences, Mashhad, Iran*; 2 *Pharmaceutical Research Centre, Department of Biotechnology, School of Pharmacy, Mashhad University of Medical Sciences, Mashhad, Iran*; 3 *Nanotechnology Research Centre, Department of Pharmaceutics, School of Pharmacy, Mashhad University of Medical Sciences, Mashhad, Iran*

**Keywords:** Crocetin, Crocus sativus, Direct precipitation, High performance liquid chromatography, Human serum samples, Saffron, Solid phase extraction

## Abstract

***Objective(s):*** The present study reports the development and validation of a sensitive and rapid extraction method beside high performance liquid chromatographic method for the determination of crocetin in human serum.

***Materials and Methods:*** The HPLC method was carried out by using a C18 reversed-phase column and a mobile phase composed of methanol/water/acetic acid (85:14.5:0.5 v/v/v) at the flow rate of 0.8 ml/min. The UV detector was set at 423 nm and 13-cis retinoic acid was used as the internal standard. Serum samples were pretreated with solid-phase extraction using Bond Elut C_18_ (200mg) cartridges or with direct precipitation using acetonitrile.

***Results:*** The calibration curves were linear over the range of 0.05-1.25 µg/ml for direct precipitation method and 0.5-5 µg/ml for solid-phase extraction. The mean recoveries of crocetin over a concentration range of 0.05-5 µg/ml serum for direct precipitation method and 0.5-5 µg/ml for solid-phase extraction were above 70 % and 60 %, respectively. The intraday coefficients of variation were 0.37- 2.6% for direct precipitation method and 0.64 - 5.43% for solid-phase extraction. The inter day coefficients of variation were 1.69 – 6.03% for direct precipitation method and 5.13-12.74% for solid-phase extraction, respectively. The lower limit of quantiﬁcation for crocetin was 0.05 µg/ml for direct precipitation method and 0.5 µg/ml for solid-phase extraction.

***Conclusion:*** The validated direct precipitation method for HPLC satisﬁed all of the criteria that were necessary for a bioanalytical method and could reliably quantitate crocetin in human serum for future clinical pharmacokinetic study.

## Introduction

Saffron, the dried stigmas of *Crocus sativus* L., is an expensive spice that is used mainly as a herbal medicine or food coloring and ﬂavoring agent in different parts of the world. Saffron originally grew in Iran, India, Spain, Greece and other countries (1). Antioxidant (2), anticonvulsant (3-7), antinociceptive, anti-inflammatory (8-10), sun protective (11, 12), antidote (13-16), DNA protective (17-18), aphrodisiac (19-20), tumoricidal and cytotoxic effects (21) were reported in the previous studies for saffron and/or its constituents.

**Figure 1:**
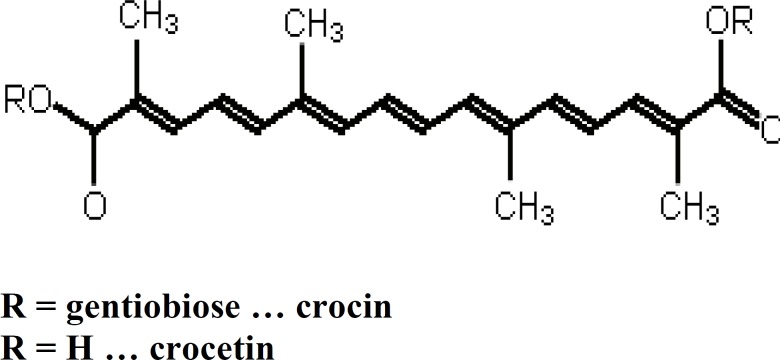
Structure of crocin and crocetin

 Saffron contains many chemical substances such as carbohydrates, vitamins, minerals, mucilage and pigments (such as carotenes and flavonoids). Crocin is the principal coloring pigment in saffron stigma. This carotenoid derivative, glycoside derivative of trans-crocetin, is one of the major biologically active components in saffron (Figure 1). Cis-crocetin and its glycosides are also present; however, these are the minor components in saffron. Crocetin is a unique carotenoid because of its short carbon chain length (20 apocarotenoid) and the two carboxyl groups at both ends of the carbon chain (22, 23). 

Different experimental studies have demonstrated a variety of pharmacological actions for crocetin such as enhancement of oxygen diffusivity (23-25), inhibition of tumor cell proliferation (26), hepatotoxicity (27) and protective effects against atherosclerosis (28). 

 Previously, some methods were developed for the analysis of crocetins using thin-layer chromatography, high-performance liquid chromatography and gas chromatography (29). On the other hand, the investigations on the pharmacological activities of crocetin were somehow obtained from the experiments *in vitro* or in animal samples. The pharmacokinetic of crocetin in rats was reported by Liu and Qian (30) and also it was indicated that orally administered crocins are converted to crocetin in mice gastrointestinal tract or during absorption (31). However, achievement of any analysis method to detect crocetin in human serum is necessary.

Therefore, the aim of present study was to propose a simple method for quantification of crocetin in human serum after protein separation by solid phase extraction or direct precipitation method. The present study reports, the development and validation of a sensitive and rapid extraction method beside high performance liquid chromatographic method for the determination of crocetin in human samples.

## Materials and Methods

***Materials***

**Figure 2:**
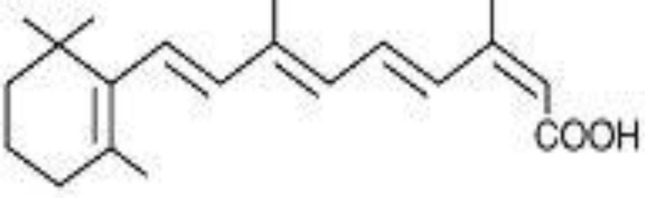
Structure of 13-cis retinoic acid

Crocetin was isolated from *C. sativus *L. in Avicenna Research Institute, Mashhad, Iran. In brief, the pigments were saponified with 10% sodium hydroxide aqueous solution at 60ºC for 4 hr. The solution was then acidified with phosphoric acid, and the yielded precipitate was washed with water. Crocetin was then crystallized from dimethylformamide. The structure and purity of standard crocetin was confirmed by UV (λ_max_: 436 and 464 nm) and ^1^H and CNMR. Methanol and acetonitrile (HPLC grade) were purchased from Caledon (Georgetown, Canada). Acetic acid, ammonium acetate and mono basic sodium phosphate were from Merck (Darmstadt, Germany). 13-cis retinoic acid, used as the internal standard (IS) (Figure 2), was obtained from Sigma-Aldrich (St. Louis, USA). 

***Human serum samples***

Five healthy volunteers with normal biochemical parameters were enrolled in this study. These subjects were taken a single oral dose of 16 mg crocetin capsule. Blood sampling was carried out at 0, 20, 40, 60, 90, 120, 150, 180 and 240 min after saffron administration. These human samples were prepared by both direct precipitation and solid phase extraction methods and were analyzed with HPLC method. The human study protocol was approved by local Ethics Committee.

***Instrumentation and chromatographic condition***

The chromatographic system was composed of a Shimadzu (Kyoto, Japan) LC-10ADVP chromatographic pump and a Shimadzu UV- SPD-10AVD spectrophotometric detector set at 423 nm. Separations were obtained on a Knauer Chromatography (Germany) C_18_ reversed-phase column (25 mm × 4 mm I.D., 5 µm) with a Knauer C_18_ guard column (5 mm×4 mm I.D., 5 µm) kept at room temperature. The mobile phase was composed of a mixture of methanol/water/acetic acid (85/14.5/0.5). The mobile phase was ﬁltered through a 0.22 µm membrane ﬁlter and degassed by an ultrasonic bath. The ﬂow rate was 0.8 ml/min and the injections were carried out through a 25 µl loop. 

 The clean-up procedure consisted of direct precipitation method or solid phase extraction. Solid phase extraction was performed using WATERS^®^ vacuum manifold system (Milford, USA) coupled to the GAST vacuum pump (Mich, USA). For extraction procedure, Cronus Bond SPE columns were used. Column size was 3 ml and ﬁlled with 200 mg silica-bond C_18_ (Cronus, UK). In order to separate the precipitated proteins, an Abbott D-37520 centrifuge (Germany) at 8500 g and 20°C was used.

***Preparation of standard solutions***

Stock solutions of crocetin and 13-cis retinoic acid as internal standard were prepared in DMSO in the concentrations of 100 and 50 µg/ml, respectively. Then, the stock solution was further diluted with blank serum and methanol: water (85:15 v/v) separately to give working solutions of 0.025, 0.05, 0.1, 0.25, 0.5, 1, 1.25, 1.75, 2.5 and 5µg/ml of crocetin. All solutions were stored at the freezer temperature (-20ºC).

***The sample preparation***

The sample preparation for the procedure was done in two different ways, direct precipitation method and solid phase extraction (SPE).

***Direct precipitation method***

This method was done according to Xi and colleagues (32) with some modifications. Briefly, the serum samples were mixed with acetonitrile containing 13-cis retinoic acid as an internal standard (1:1 v/v). Then, the mixtures were shaken in the vortex mixer for 30 sec and centrifuged for 15 min at 8500 g. The obtained supernatant was centrifuged at 8500 g for another 5 min and then transferred into chromatographic vials, from where 25 μl of solution was injected into the column.

***Solid phase extraction (SPE)***

Serum sample (50 µl) was mixed with 50 µl of 0.1 mol/l sodium phosphate buffer (pH 7.0) and 0.5 ml of methanol containing 13-cis retinoic acid as an internal standard. The mixture was vortexed for 1 min and centrifuged at 8500 g for 10 min. A portion of supernatant (0.4 ml) was mixed with 1.0 ml of 0.2% ammonium acetate aqueous solution. The mixture was then applied to a Bond Elut C_18_ (200 mg) solid phase extraction cartridge (Cronus, UK), which was washed with methanol (1.0 ml) and equilibrated with water/methanol (3:1, v/v) containing 0.2% ammonium acetate (2.0 ml) before use. After the sample was loaded, the cartridge was washed with water/methanol (3:1, v/v) containing 0.2% ammonium acetate (2.0 ml), 0.2% ammonium acetate aqueous solution (2.0 ml) and hexan (2.0 ml), respectively. After that methanol (1.0 ml) was loaded, the eluate was collected. The methanolic eluate was evaporated to dryness under a stream of nitrogen at room temperature. The residue was dissolved in 150 µl water:methanol (1:1 v/v). Then, the mixtures were shaken in the vortex mixer for 1min and centrifuged for 8 min at 8500 g. The obtained supernatant was transferred into chromatographic vials, from where 25 μl of solution was injected into HPLC system for analysis (30).

***Method validation***

*Linearity and range* *(* *calibration curves* *)*

Linearity of the analytes was evaluated using freshly prepared samples covering the analysis range. The calibration curve of crocetin was constructed by plotting the peak area ratios of the respective analyte to the internal standard (y) against the analyte concentration (x) (expressed as µg/ ml). The procedure was carried out in triplicate for each concentration. The calibration curve follows the equation of y = bx + a, where b and a refer to the slope and y-intercept, respectively. The analyte concentration, x, was determined from the calibration curve using the formula: x = (y − a)/b 

 The lowest concentration of the analytes with an accuracy of 80–120%, as well as the coefficient of variation of <20% was regarded as the lowest limit of quantiﬁcation (LLOQ). This concentration was used as the ﬁrst calibration point for the calibration curve.

 The applied requirements for a valid calibration model were: a regression coefficient higher than 0.990 (R^2^), and the residuals and coefficient of variation (CV%) to be within ±20% at the lower limit of quantification (LLOQ) and ± 5% for the rest of the concentrations tested.

*Selectivity*

Drug-free serum samples were processed using the sample preparation and analytical procedures mentioned above. Selectivity of the method was evaluated by examining the extent to which endogenous substances and degradation products could possibly interfere with the retention time of the analyte.

*Accuracy and precision*

The accuracy of the method was determined by comparing the mean measured concentration with the nominal concentrations of the crocetin in all concentration levels. Five replicates of each of the concentrations were analyzed. The accuracy was acceptable if the ratio of the difference between the mean measured concentration and the nominal values against nominal concentration (bias%) did not exceed±15%.

 Intraday precision was evaluated by extracting and analyzing five replicates of a sample within the same run in the same day. This was done in all concentrations. The interday precision was evaluated by analyzing a sample in all concentrations on 5 different days. The requirement for precision was acceptable if the coefficient of variation (CV%) did not exceed±15%, either for intraday or inter days assay.

*Extraction recovery*

The relative recovery of the samples was determined by the following equation:

$\mathrm{Relative}\mathrm{recovery}=\frac{\mathrm{serum crocetin mean peak area ratios}/\mathrm{serum 13cis retinoic acid mean peak area ratios}}{\mathrm{methanolic crocetin mean peak area ratios}/\mathrm{methanolic 13cis retinoic acid mean peak area ratios}}$

*Resolution*

The resolution (R_s)_ two components is a measure of how well they are separated. It was defined by the following equation (B was the later eluting peak):

$\mathrm{Rs}=\frac{2(\mathrm{tR peak A}-\mathrm{tR peak B})}{\mathrm{sum of the peak widths at base in units of time}}$

In practice, a resolution factor of 1.5 was considered the lowest practical value for quantitative analysis (32).

*Symmetry factor*

Peak symmetry (As) may be calculated by determining the ratio of the distances between the peak maximum and the leading and trailing edges at 10% of peak height. Values of as of between 0.85 and 1.3 were generally acceptable (33).

**Figure 3:**
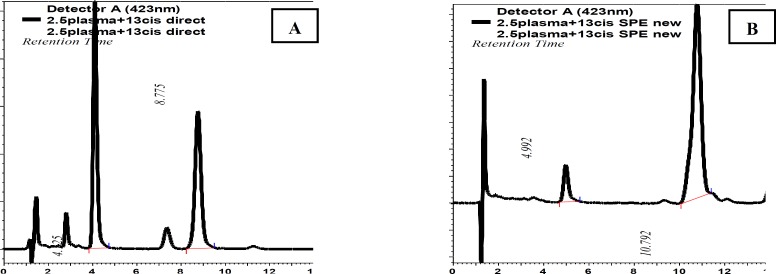
HPLC chromatogram of crocetin and 13-cis retinoic acid prepared by (A) direct precipitation (B) solid phase extraction

## Results

Figure 3 shows the representative chromatograms of human serum containing crocetin and 13-cis retinoic acid (IS) which extracted with two different methods (direct precipitation or solid phase extraction), obtained from the HPLC-UV system. The retention times for crocetin and 13-cis retinoic acid were respectively 4.125 and 8.775 min after preparation of samples by direct precipitation method whereas these times were shifted to 4.992 and 10.792 min after using the solid phase extraction for preparation of samples. 

***Linearity and range***

The calibration curve of crocetin was linear from 0.05 to 1.25 µg/ml serum for direct precipitation method and 0.5 to 5 µg/ml serum for solid phase extraction method. Their regression coefficients (R^2^) were 0.999 and 0.990 for direct precipitation method and solid phase extraction methods, respectively.

 The LLOQ of crocetin was 0.05 µg/ml serum for direct precipitation method with the coefficient of variation (CV) of 2.6% and 0.5 µg/ ml serum for solid phase extraction method with the coefficient of variation (CV) of 5.34%.

***Selectivity***

**Figure 4:**
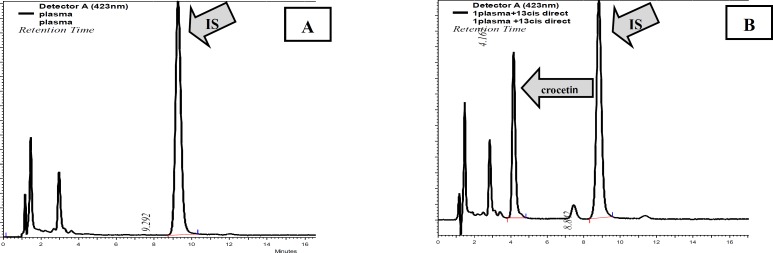
HPLC chromatogram of (A) extracted blank (drug-free) human serum containing 13-cis retinoic acid (IS); (B) extracted human serum containing crocetin and 13-cis retinoic acid (IS)

Both extraction methods with HPLC used for determining crocetin were found to be selective. No endogenous substances or interfering peaks were observed at the retention time of the crocetin. Figure 4 shows the representative chromatograms of extracted blank human serum containing 13-cis retinoic acid (IS) and the serum extract which contained crocetin and 13-cis retinoic acid (IS) obtained from the HPLC-UV system. 

**Table 1:** Accuracy of the determination of crocetin in human serum (n= 5)

Direct precipitation		Solid phase extraction		Bias (%)		Accuracy	
C_nominal_ (µg/ml)	C _measured_ (µg/ml)	C_nominal_ (µg/ml)	C _measured_ (µg/ml)	Direct precipitation	Solid phase extraction	Direct precipitation	Solid phase extraction
0.05	0.0496	0.5	0.6171	-0.83	+23.42	99.17	76.58
0.1	0.0909	1	1.1333	-9.091	+13.33	90.91	86.67
0.25	0.2562	1.25	1.2625	2.48	+0.9988	102.48	99.00
0.5	0.5041	1.75	1.567	0.83	-10.45	100.83	110.45
1	1	2.5	2.3031	0	-7.88	100	107.88
1.25	1.2479	5	5.137	-0.17	+2.77	99.83	97.26
							

***Accuracy and precision***

The accuracy of the method with direct precipitation ranged between -9.09 and 2.48 and with solid phase extraction ranged between -23.42 and 10.45. The results are given in Table 1. The intraday coefficient of variations for crocetin in direct precipitation method was less than 3% for all the concentrations and in solid phase extraction method was less than 5.5%, while the interday coefficient of variations for crocetin was less than 6.5% for all the concentrations and in solid phase extraction was less than 12.8% as indicated in Table 2.

***Extraction recovery***

The mean extraction recovery of the crocetin was somehow good in both methods (direct precipitation 55-88% and solid phase extraction 47-69 %). The data are shown in Table 3.

***Resolution***

The resolution factors for direct precipitation and solid phase extraction methods were 4.2 and 4.65, respectively.

***Symmetry factor***

In direct precipitation method, the symmetry factors for crocetin and 13-cis retinoic acid were 1.04 and 0.93 while in solid phase extraction method were 0.85 and 0.88, respectively. 

**Table 2:** Precision of the determination of crocetin in human serum (n= 5)

Direct precipitation			Solid phase extraction		
C_nominal_ (µg/ml)	Intraday CV (%)	Interday CV (%)	C_nominal_ (µg/ml)	Intraday CV (%)	Interday CV (%)
0.05	2.6	4.08	0.5	5.43	5.79
0.1	0.48	6.03	1	0.64	12.74
0.25	0.37	3.74	1.25	4.81	5.13
0.5	1.52	1.69	1.75	4.59	5.91
1	0.66	3.56	2.5	3.88	0.02
1.25	0.74	3.52	5	1.01	5.65

**Table 3:** Extraction relative recovery of crocetin in human serum

Direct precipitation		Solid phase extraction	
C_nominal _(µg/ml)	Relative Recovery (%)	C_nominal _(µg/ml)	Relative Recovery (%)
0.05	61	-	-
0.1	60	-	-
0.25	55	-	-
0.5	58	0.5	58
1	77	1	69
1.25	75	1.25	47
1.75	88	1.75	64
2.5	73	2.5	57
5	77	5	64

**Figure 5:**
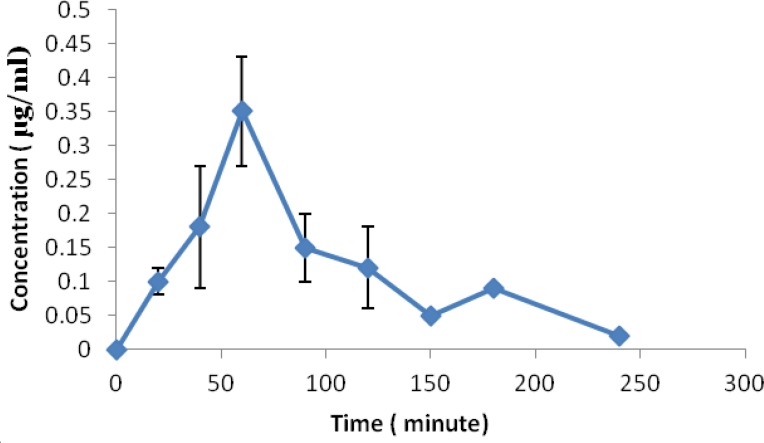
Serum concentrations vs. time profiles of crocetin after oral administration of single 16 mg crocetin (Mean±SD, n=5)

***Human samples***

Figure 5 shows the concentration of crocetin in human samples in different intervals.

## Discussion

As it was mentioned previously, establishment of validated extraction and analysis method for human serum samples is important for clinical pharmacokinetics studies of crocetin. For this reason, in the present study, extraction method beside HPLC method was developed and validated.

 In this study, 13-cis retinoic acid was used as internal standard. This compound was selected as the internal standard (IS) due to the similarity of its structure to that of the crocetin and its suitable chromatographic properties. According to the International Conference on Harmonization (ICH-1996), the internal standard should be structurally similar to the main compound of analysis (34). Also, 13-cis retinoic acid was well resolved with a baseline separation from crocetin, and the analysis was completed within 12 min. 

 Previously, mixture of methanol:water:acetic acid (75:24.5:0.5) was used as mobile phase (30) but in this study, the mobile phase was consisted of a mixture of methanol:water:acetic acid (85:14.5:0.5) to decrease the retention time.

 We compared two different extraction methods (Direct precipitation and Solid phase extraction) for detection of crocetin in human serum by HPLC. Comparison of these methods followed by HPLC, the direct precipitation method had higher extraction yields (>70% versus 59%), better sensitivity (LLOQ = 0.05 µg/ml versus 0.5 µg/ ml) and better linearity (0.999 versus 0.990), thus enabling the compounds to be analyzed at very low concentration levels. The differences in LLOQs may be related to the higher drug loss by SPE method compared to direct precipitation method. Direct precipitation method involves one simple step while in SPE method elution and passage of samples through solid phase influence on limit of detection of analysis method. 

 Accuracy and precision of both methods were found to be within the acceptable range (except the first concentration of solid phase extraction), but direct precipitation showed a better result. The use of direct precipitation method poses several advantages with respect to the solid phase extraction procedures. In fact, the proposed procedure is simple, faster and requires lower volumes of organic solvents. Furthermore, direct precipitation method had a wide linearity range. Also, this method does not involve laborious and time-consuming sample preparation.

 Previously, high dose of crocetin was used to evaluate the pharmacokinetics of crocetin in animal models. In the study of He *et al *(28), 50 mg/kg was administrated or in Zheng *et al* study (35), 15 mg/kg crocetin was added to the rabbit food. As lower dose of crocetin can be administrated in the human studies compared to animal studies (0.5 mg/kg vs 50 mg/kg), lower concentration of crocetin expected in human samples. Consequently, it seems development of precise method for human pharmacokinetics study is necessary. Umiga *et al *evaluated the pharmacokinetic profile of crocetin in healthy adult human volunteers and used a solid phase extraction for assays of plasma crocetin but in this study we demonstrated the direct precipitation method was better for analysis of serum crocetin concentration (36). After administration of 16 mg crocetin by healthy volunteers, crocetin concentration range was 0.09-0.35 µg/ml at different sampling intervals. Umiga *et al *reported mean peak serum concentration 0.2 µg/ml after single oral dose 15 mg crocetin in healthy volunteers (36). These concentration ranges show that the reported method is valid for quantitation of crocetin in human sample and applicable for pharmacokinetic studies. In the previous studies, validation parameters were not calculated but in the present study, some parameters were calculated and reported for validation of methods. Dimitra *et al *reported a SPE method for monitoring crocetin in human plasma (37). This study reported a simple validated method without requirement to solid phase extraction for determination of serum concentration of crocetin in human.

## Conclusions

Analytical data of the present study can be used to analyse the concentration of crocetin for pharmacokinetics study. The validated direct precipitation method followed by HPLC method satisﬁed all the criteria that were necessary for a bioanalytical method. The presented extraction method was simple and involved only one step.
